# Pharmacological activity of OST-01, a natural product from baccharis coridifolia, on breast cancer cells

**DOI:** 10.1186/s13045-025-01668-4

**Published:** 2025-02-07

**Authors:** HyunJun Kang, Dinh Hoa Hoang, Melissa Valerio, Khyatiben Pathak, William Graff, Alexis LeVee, Jun Wu, Mark A. LaBarge, David Frankhouser, Russell C. Rockne, Patrick Pirrotte, Bin Zhang, Joanne Mortimer, Le Xuan Truong Nguyen, Guido Marcucci

**Affiliations:** 1 Department of Hematologic Malignancies Translational Science, Beckman Research Institute and City of Hope National Medical Center, Duarte, CA USA; 2 Early Detection and Prevention Division, Translational Genomics Research Institute, Phoenix, AZ USA; 3 Ostentus Therapeutics, Inc, Newport Coast, CA USA; 4 Division of Medical Oncology and Experimental Therapeutics, Beckman Research Institute and City of Hope National Medical Center, Duarte, CA USA; 5 City of Hope Comprehensive Cancer Center, Duarte, CA USA; 6 Department of Population Sciences, Beckman Research Institute and City of Hope National Medical Center, Duarte, CA USA; 7 Department of Computational and Quantitative Medicine, Division of Mathematical Oncology, Beckman Research Institute and City of Hope National Medical Center, Duarte, CA 91010 USA

**Keywords:** TNBC, BCSC, Natural product, OST-01, Ferroptosis

## Abstract

Natural products have long been a viable source of therapeutic agents, providing unique structures and mechanisms that may be beneficial for cancer treatment. Herein we first report on the anticancer activity OST-01, a natural product from Baccharis Coridifolia, on breast cancer cells, including triple-negative breast cancer (TNBC). OST-01 significantly inhibited cell proliferation and oncogenic activities of TNBC cells in vitro. OST-01 also markedly inhibited TNBC tumor growth in vivo, with > 50% reduction in tumor size compared to vehicle control treatment in different in vivo models, i.e., cell line-derived (CDX), patient-derived (PDX), and mammary fat pad xenografts. Mechanistically, OST-01 induces ferroptosis by downregulating LRP8-regulated selenoproteins, i.e., GPX4. A shift from a basal-mesenchymal to a luminal-epithelial state of breast cancer stem cells (BCSCs) as supported by the downregulation of stemness (e.g., CD44) and mesenchymal (e.g., FN1 and vimentin) markers, along with the upregulation of differentiation markers (e.g., CD24) and luminal-epithelial markers (e.g., CK19), was also observed.

**Supplementary Information:**

The online version contains supplementary material available at 10.1186/s13045-025-01668-4.

**To the Editor**,

Natural products (NPs) have been essential in cancer drug discovery, with noteworthy examples of plant-derived chemotherapeutics currently part of stand-of-care regimens [1]. Baccharis (B) coridifolia, a South American plant of the Asteraceae family, is used in botanical medicine without reported toxicity in humans [2]. We previously showed that OST-01, a NP derived from B. coridifolia, has in vivo efficacy in acute myeloid leukemia (AML) by disrupting c-Myc-dependent ribogenesis of leukemic stem cells [3]. Herein we report that OST-01 is also cytotoxic to a variety of solid tumors, including lung, colon, ovarian, glioblastoma, pancreatic, and breast cancers (Sup. Figure S1A). To this end, we investigated the activity of OST-01 in breast cancer (BC), including triple-negative breast cancer (TNBC), an aggressive subtype enriched with breast cancer stem cells (BCSCs) [4–6].

OST-01 effectively inhibited proliferation of triple-positive BT474 and TNBC (MDA-MB-231, MDA-MB-468, BT549, and 4T1) cells, with IC50 values up to 3.8 µL/mL (Sup. Figure S1B). At 1 µL/mL, OST-01 also suppressed proliferation and induced apoptosis in patient-derived TNBC cells while sparing normal human mammary epithelial cells (HMECs, Fig. 1A-B). Of note, the pro-apoptotic activity of OST-01 in triple-positive and TNBC cells appeared stronger than that of other standard-of-care chemotherapeutics (i.e., Taxol and 5-FU), used at concentrations known to demonstrate in vitro activity in inducing apoptosis in cancer cells (Fig. 1C and Sup. Figure S1C). OST-01 also inhibited colony formation, anoikis resistance, wound healing, and invasion (Fig. 1D and Sup. Figure 2 A-D), reduced spheroid formation, and increased TNBC cell death in 3D cultures (Sup. Figure 2E). The in vivo activity of OST-01 was tested in four murine models: a triple-positive BC CDX (BT474), a TNBC CDX (MDA-MB-231), a TNBC PDX, and a luciferase-expressing MDA-MB-231 fat pad xenograft. Mice received either vehicle or OST-01 (1 µL/g, BID) via oral gavage starting 14 days post-injection. After 5 weeks, OST-01 reduced tumor volumes by at least 50% across all models, with p-values of 0.0024 for the BT474 CDX, 0.0019 for the MDA-MB-231 CDX, and 0.0017 for the TNBC PDX (Fig. 1E-G). In the fat pad model, bioluminescence imaging and tumor measurements confirmed approximately 50% reduction in tumor size and weight (*p* = 0.0002, Fig. 1G), validating OST-01’s in vivo efficacy.

**Fig. 1:**
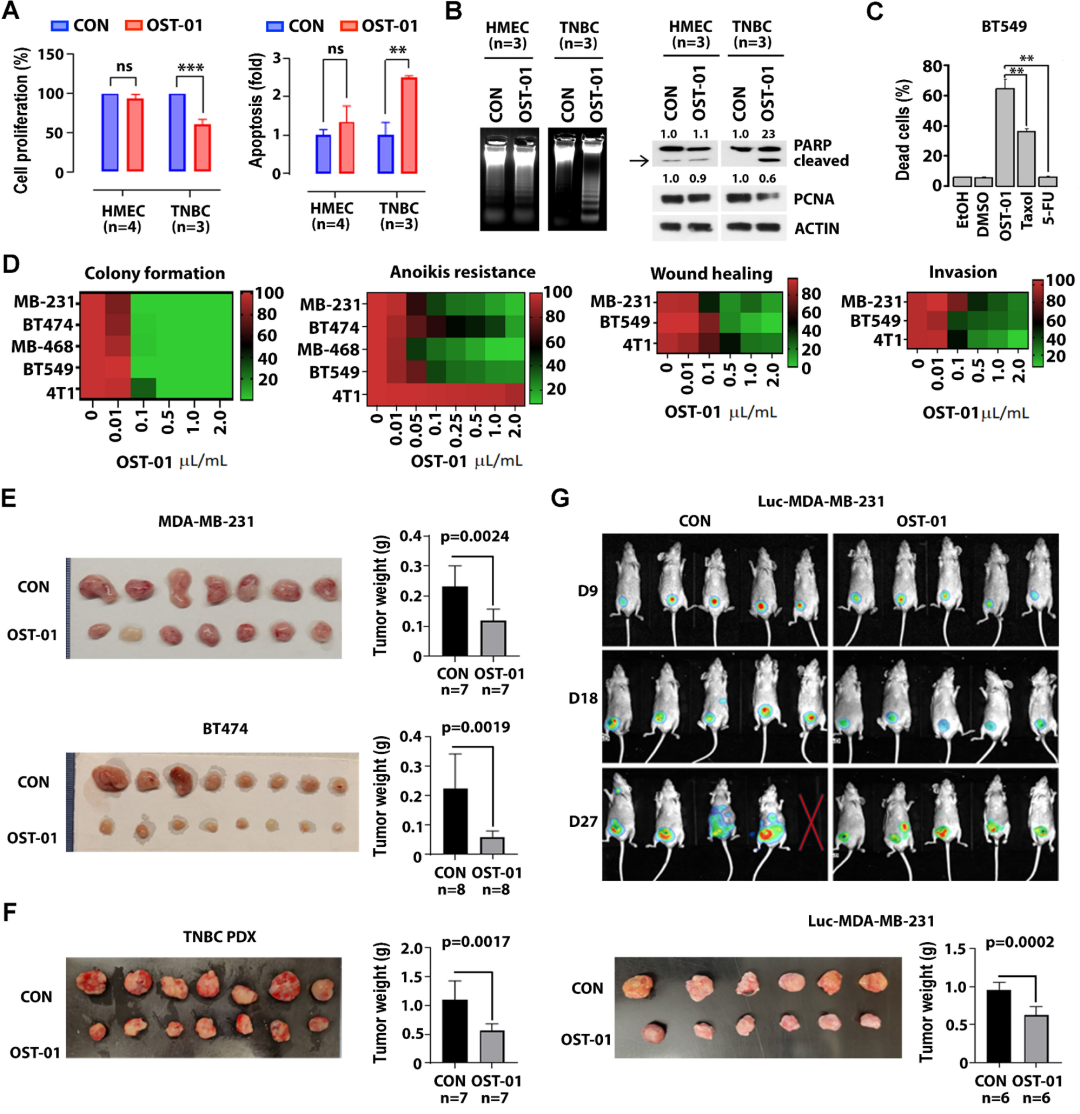
Effects of OST-01 on oncogenic activities in TNBC cells in vitro and tumor growth in vivo. **A-B** Effects of OST-01 on proliferation and apoptosis in primary HMEC and TNBC cells. Primary HMEC (*n* = 4) and TNBC (*n* = 3) cells were treated with 1 µL/mL of ethanol control or OST-01 (1 µL of OST-01 extract contains 250 µg of dry plant extract) for 24 h. **A** Levels of cell proliferation (left) and apoptosis (right). **B** DNA fragmentation (left) and levels of PCNA and PARP cleavage (right). Quantification of protein expressions are shown on top. **C** Comparative effects of OST-01 and other therapeutic drugs on BT549 TNBC cell lines. The cells were treated with 1 µL/mL of OST-01 or 2 µM of Taxol or 5-FU for 24 h. Apoptosis was measured by Annexin V staining. **D** Effects of OST-01 on oncogenic activities in TNBC cells. The indicated triple-positive BC cell line (BT474) and TNBC cell lines [MDA-MB-231 (MB-231), MDA-MB-468 (MB-468), 4T1, and BT549] were treated with increasing doses of OST-01 for 24 h. From left to right: heatmap displaying colony formation, anoikis resistance, wound healing, and invasion levels. **E-G** Effects of OST-01 on TNBC tumor growth in vivo. **E** MDA-MB-231 TNBC cells or BT474 triple-positive BC cells (10^6^ cells) were subcutaneously injected into nude mice. After 2 weeks, the transplanted mice were treated with ethanol control or OST-01 (1 µL/g, oral gavage, BID) for 5 weeks. Left, image of isolated tumors. Right, tumor weights (MDA-MB-231: each group, *n* = 7; BT474: each group, *n* = 8). **F** Primary TNBC-PDX tumors (HCI-023) were subcutaneously transplanted into NSG mice. After 2 weeks, the transplanted mice were treated with ethanol control or OST-01 (1 µL/g, oral gavage, BID) for 5 weeks. Left, image of isolated tumors. Right, tumor weights (each group, *n* = 7). **G** Mammary fat pad model using Luc-MDA-MB-231 TNBC cells. 10^6^ luciferase (Luc)-expressing MDA-MB-231 cells were injected into the mammary fat pad of NSG mice (each group, *n* = 10). After 7 days, the mice were treated with ethanol control or OST-01 (1 µL/g, oral gavage, BID). Top, luminescence images taken on days 9, 18, and 27 after treatment. Bottom, image of isolated tumors (left) and tumor weights (right) (each group, *n* = 6)

To investigate OST-01’s anticancer mechanism, we performed RNA sequencing on BT474 (triple-positive) and two TNBC cell lines (4T1, MDA-MB-231) treated with ethanol control or OST-01 (1 µL/mL). OST-01 upregulated genes involved into inflammatory response and TNFα/IL6/JAK/STAT3 signaling, while downregulating those related to cell cycle, ROS, mTORC, and Myc pathways (Sup. Figure 3 A-B). OST-01 notably downregulated BCSC-associated genes, including LRP8, RAD54L, STC-1, SDC-1, and SRSF3 (Fig. 2A-B and Sup. Figure 3 C-E), indicating its targeted action on BCSCs.

**Fig. 2:**
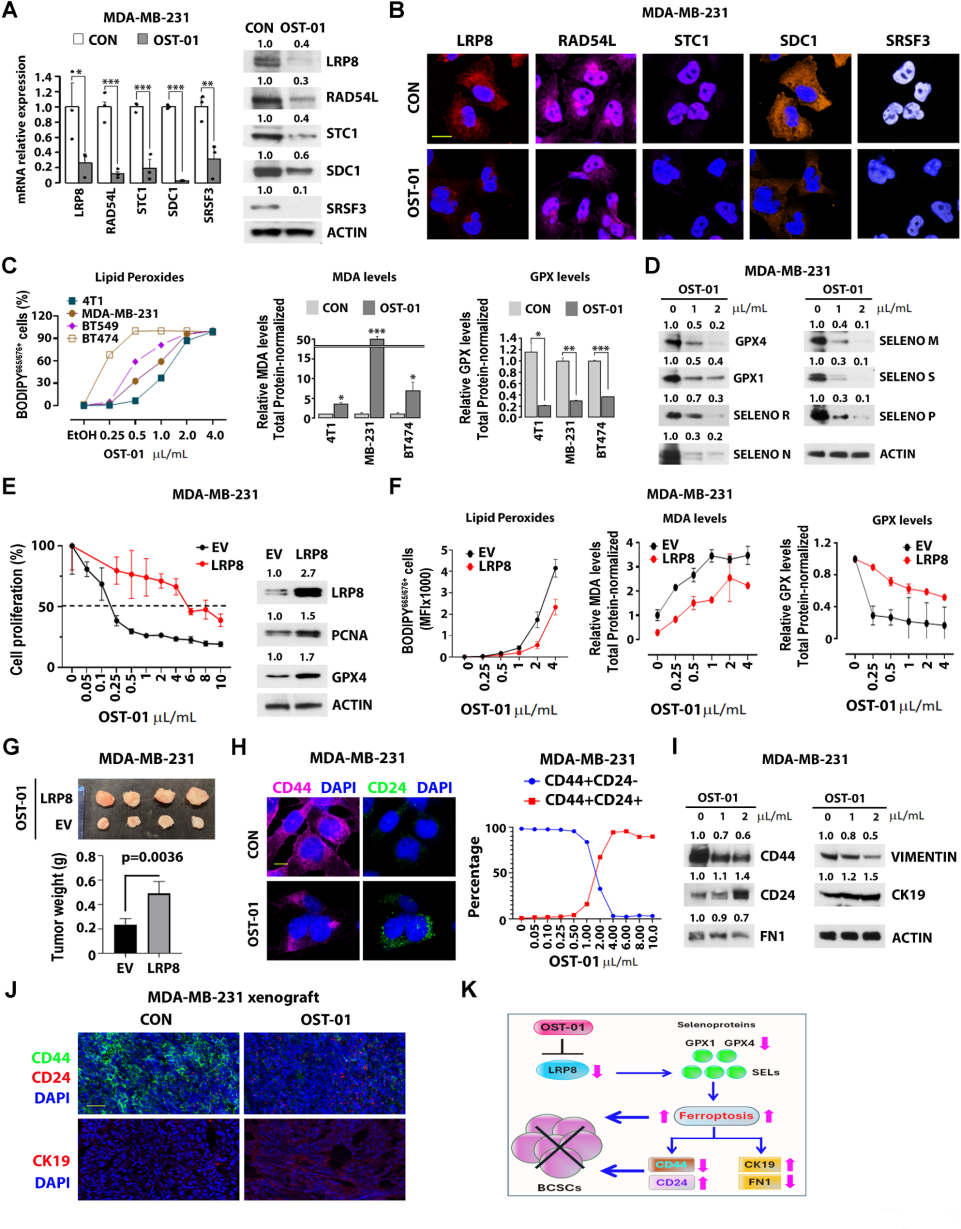
Effects of OST-01 on LRP8-regulated ferroptosis in TNBC cells. **A-B** Effects of OST-01 on the expression of key regulators of BCSC. MDA-MB-231 cells were treated with 1 µL/mL of ethanol control or OST-01 (1 µL of OST-01 extract contains 250 µg of dry plant extract) for 24 h. **A** Left, mRNA levels were measured by qPCR. Right, protein levels were measured by immunoblotting. Quantification of protein expressions are shown on top. **B** The treated cells were stained with the indicated antibodies; representative confocal images are shown. Scale bar, 10 μm. **C** Effects of OST-01 on ferroptosis in TNBC cells. The indicated TNBC cell lines (4T1, MDA-MB-231, and BT549) and the triple-positive BC cell line BT474 were treated with OST-01 in a dose-dependent manner for 24 h. Left: Lipid peroxide levels were measured using a lipid peroxidation assay. Middle: Malondialdehyde (MDA) levels were assessed. Right: Glutathione peroxidase (GPX) levels were measured by a GPX activity assay. **D** Effects of OST-01 on selenoprotein expression in TNBC cells. MDA-MB-231 TNBC cells were treated with OST-01 in a dose-dependent manner for 24 h. Cell lysates were immunoblotted with the indicated selenoprotein antibodies. Quantification of protein expressions are shown on top. **E-G** Effects of LRP8 overexpression on OST-01-induced ferroptosis and TNBC cell growth inhibition. **E** MDA-MB-231 TNBC cells with empty vector control or LRP8 overexpression were treated with OST-01 in a dose-dependent manner for 24 h. Left, cell proliferation was assessed. Right, immunoblotting with the indicated antibodies was performed. Quantification of protein expressions are shown on top. **F** MDA-MB-231 TNBC cells with empty vector control or LRP8 overexpression were treated with OST-01 in a dose-dependent manner for 24 h. Levels of ferroptosis were measured. Left, lipid peroxides levels. Middle, MDA levels. Right, GPX levels. **G** 10^6^ MDA-MB-231 TNBC cells with empty vector control or LRP8 overexpression were subcutaneously injected into nude mice. After 2 weeks, the transplanted mice were treated with OST-01 (1 µL/g, oral gavage, BID) for 5 weeks. Left, image of tumors. Right, tumor weight (each group, *n* = 4). **H** Effects of OST-01 on CD44 and CD24 expression. MDA-MB-231 TNBC cells were treated with 1 µL/mL of ethanol control or OST-01 for 24 h. Left, the treated cells were stained with anti-CD44 (pink) and anti-CD24 (green) antibodies. Images were captured using confocal microscopy. Scale bar, 10 μm. Right, graph showing the changes in CD44 + CD24- and CD44 + CD24 + populations upon OST-01 treatment. **I-J** Effects of OST-01 on BCSCs in vitro and in vivo. **I** MDA-MB-231 TNBC cells were treated with OST-01 in a dose-dependent manner for 24 h. Cell lysates were immunoblotted with the indicated antibodies. Quantification of protein expressions are shown on top. **J** MDA-MB-231 xenograft tumors isolated from mice treated with ethanol control or OST-01 (as described in Fig. 1E) were subjected to frozen sectioning and stained with the indicated antibodies. Images were captured using confocal microscopy. Scale bar, 1 μm. **K** Schematic model of the mechanism of action of OST-01 on TNBC cells. OST-01 inhibits LRP8 expression, leading to reduced levels of selenoproteins, including GPX4. The decrease in selenoproteins induces ferroptosis, which in turn suppresses the stemness activities of BCSCs. This effect is achieved through the induction of mesenchymal-to-epithelial transition, resulting in enhanced differentiation of the cells

Focusing on TNBC cells, we observed that OST-01 downregulated LRP8 (ApoER2), a key regulator of ferroptosis—a process crucial for cancer cell survival, characterized by lipid peroxidation, elevated malondialdehyde (MDA), and decreased GPX4 activity [7, 8]. LRP8 promotes translation of selenoproteins (SELs) like GPX4 by promoting selenium uptake [9–12]. Increased SEL production prevents ferroptosis and supports cancer cell homestasis [7, 8]. coessentiality network analysis of the RNA-seq data from OST-01-treated TNBC cells showed the association of LRP8 with SELs, selenocysteine metabolism, and ferroptosis (Sup. Figure 4 A). Gene ontology linked LRP8 downregulation to oxidative stress and glutathione metabolism (Sup. Figure 4B). OST-01 treatment led to increased lipid peroxidation, elevated MDA levels, and decreased GPX4, (Fig. 2C), along with reduced SELs expression (Fig. 2D and Sup. Figure S5**A**), confirming triggered ferroptosis. In vivo, BC cells from OST-01-treated xenografts showed lower SDC1, LRP8, and GPX4 levels than controls (Sup. Figure S5**B**). Additionally, LRP8 overexpression (OE) in MDA-MB-231 TNBC cells conferred resistance to OST-01, increasing proliferation and reducing ferroptosis (Fig. 2E-F) and resulting in larger tumors in xenografts (Fig. 2G), supporting LRP8’s role in OST-01-induced ferroptosis. Of note, GSEA indicates that OST-01 treatment promotes a shift in TNBC and triple-positive BC cells from a basal-mesenchymal to a luminal-epithelial state, with reduced CD44 and increased CD24 expression in MDA-MB-231 cells (Fig. 2H-J and Sup. Figure 6). To confirm these findings, we showed that OST-01 reduced mesenchymal markers (FN1, vimentin) and increased CK19, a luminal-epithelial marker (Fig. 2I-J).

In conclusion, we first report on the activity of a novel natural product, OST-01, on TNBC. OST-01 induces ferroptosis by downregulating LRP8 and affecting key pathways related to BCSCs (Fig. 2K), demonstrating potential for overcoming resistance mechanisms in TNBC. The OST-01 unique activity to target BCSC-associated pathways and induce LPR8-dependent ferroptosis along with an expect safe toxicity profile (based on the phytomedical use of B. coridifolia in South America) underscores its clinical potential. Further investigations into mechanisms of action and synergistic combinations with other therapeutics are warranted to address treatment-resistant challenges encountered by current treatment regimens.

## Electronic supplementary material

Below is the link to the electronic supplementary material.

Supplementary Material 1

Supplementary Material 2

Supplementary Material 3

Supplementary Material 4

Supplementary Material 5

Supplementary Material 6

Supplementary Material 7

Supplementary Material 8

## Data Availability

No datasets were generated or analysed during the current study.

## Declarations

For animal studies, all mice were maintained in an Association for Assessment and Accreditation of Laboratory Animal Care (AAALAC) accredited facility, and all experimental procedures adhered to federal and state government guidelines as well as institutional protocols approved by the Institutional Animal Care and Use Committee at City of Hope (IACUC #22043).

## Abbreviations

AML: Acute Myeloid Leukemia
LSCs: Leukemia stem cells
TNBC: Triple-negative breast cancer
BCSC: Brease cancer stem cells
SEL: Selenoproteins
NP: Natural products
HER: Human epidermal growth factor receptor
ROS: Reactive oxygen species
MOA: Mechanism of action
GSEA: Gene set enrichment analysis
PCNA: Proliferating cell nuclear antigen
HSC: Hematopoietic stem cell
MNC: Mononuclear cell
HMEC: Human mammary epithelial cell
PDX: Patient-derived xenograft
WST-1: Water-soluble tetrazolium salt
FDR: False discovery rate
NES: Normal enrichment score
