# Supplementary material for: Pharmacological activity of OST-01, a natural product from baccharis coridifolia, on breast cancer cells
Source: J Hematol Oncol. 2025 Feb 7;18:16. doi: 10.1186/s13045-025-01668-4 (PMC11806613; doi:10.1186/s13045-025-01668-4)
Supplement: Supplementary file 1 — Supplementary Material 1 [file 13045_2025_1668_MOESM1_ESM.docx]

**SUPPLEMENTAL INFORMATION**

1. **Supplementary Materials and Methods** (Page 2 to Page 7)
2. **Supplementary References:** (Page 7 to Page 8)
3. **Supplementary Tables:** (Page 9)

**+ Supplementary Table S1.** List of antibodies used for IP, IB, and IF analysis

1. **Supplementary Figures Legends** (Page 10 to Page 13)

+ **Sup. Figure S1.** Effects of OST-01 on proliferation and apoptosis in human cancer cell lines

+ **Sup. Figure S2.** Effects of OST-01 on oncogenic activities in TNBC cells in vitro

+ **Sup. Figure S3.** Effects of OST-01 on LRP8 signaling in TNBC cells

+ **Sup. Figure S4.** Effects of OST-01 on LRP8-regulated ferroptosis in TNBC cells

+ **Sup. Figure S5.** Effects of OST-01 on the expression of LRP8 and selenoproteins in TNBC cells

+ **Sup. Figure S6.** Effects of OST-01 on mesenchymal-epithelial transition in TNBC cells

+ **Sup. Figure S7.** Raw Western Blot data.

1. **Supplementary Figures**

**Supplementary Materials and Methods**

**OST-01 Manufacture**

OST-01 is a natural product produced by Ostentus Therapeutic Incorporation (1). To prepare OST-01, leaves and stems from Baccharis coridifolia were harvested, washed in distilled water, dried with paper towels, and cut into small pieces. One kilogram of plant material was macerated with 4 liters of 95% ethanol in the dark at 15–20°C for 30 days. The pressed extract was then filtered, yielding an eluent with a concentration of 250 mg/mL. This extract, comprising one or more anti-oncogenic phytochemicals, was designated as OST-01 (patent #UEOST1-0001US). For each microliter (1 µL) of OST-01 used in the study, 250 µg of dry extract is present.

**Cell cultures, Plasmid and Chemicals**

Cancer cell lines using in this study were purchased from the American Type Culture Collection and maintained in DMEM (Dulbecco’s Modified Eagle Medium), IMDM (Iscove’s Modified Dulbecco’s Medium) or RPMI (Roswell Park Memorial Institute) medium supplemented with 10% FBS and 100 units of penicillin/streptomycin at 37°C with 5% CO_2_ and high humidity. Human cell lines purchased from ATCC more than 6 months prior to submission of this manuscript and not frozen at an early passage were authenticated using ATCCs’ human short tandem repeat (STR) DNA profiling authentication service. Morphology of cell lines was monitored routinely, and cell lines were routinely subjected to mycoplasma detection using a mycoplasma detection kit (Roche, Germany).

Flag-HA-LRP8 was a gift from Dr. Peter Ten Dijke (2).

Taxol (Paclitaxel, Cat# HY-B0015) and 5-FU (Cat#HY-90006) were purchased from MedChemExpress (MEC).

**Cell proliferation assay**

A water-soluble tetrazolium salt (WST-1; Cat #5015944001, Millipore Sigma) assay was performed to quantify the anticancer effects of OST-01 on cancer cell growth. Cells were plated at density of 15,000 cells per well of 96-well plate and treated with different dose of OST-01 for 24 hours in 37°C incubator. Then, 10 μL of WST-1 solution was added to 100 μL of culture media and the plate was incubated at 37°C incubator for 2 to 3 hours. The metabolic product of WST-1, Formazan, was measured using a multi-well spectrophotometer (wavelength, 450 nm).

**Comparing Effects of OST-01, Taxol, and 5-FU on TNBC Cells**

TNBC cells were treated with 1 µL/mL of OST-01, 2 µM of Taxol, or 2 µM of 5-FU for 24 hours, and apoptosis was assessed using Annexin V staining. Given that OST-01 contains multiple active compounds at varying concentrations, its concentration was expressed as mg dry extract per mL (1 µL of OST-01 corresponds to 250 µg of dry plant extract) rather than in molar terms.

The concentration of Paclitaxel (2 µM) was chosen based on its documented ability to exhibit inhibitory effects on TNBC cells at micromolar levels during shorter treatment durations, such as 24 or 48 hours (3-6), even though its IC50 is typically in the nanomolar range for longer treatment periods (72 hours) (7-10). Similarly, 2 µM of 5-FU was selected based on its demonstrated in vitro activity in inducing apoptosis in cancer cells (11, 12). These concentrations were selected to ensure experimental consistency and to provide a meaningful comparison of the pro-apoptotic effects of OST-01 relative to standard chemotherapeutics.

**Colony forming assay**

To assess colony-forming capacity, 100 cells were plated in each well of a 6-well plate. After three to five days, once the cells began forming small colonies, they were treated with either ethanol or OST-01 at indicated concentration (µL/mL, v/v). Following two weeks of incubation, the colonies were stained with crystal violet to enhance visualization and quantification.

**Invasion assay**

The cell invasion assay was performed using the Cell Invasion Assay Kit (ECM550m, EMD Millipore Corp) following the manufacturer’s instructions. Briefly, cells were plated at a density of 7.5 x 10⁴ cells per insert, which contained a polycarbonate membrane with an 8.0 µm pore size, along with 300 µL of serum-free media. To the lower chamber, 500 µL of culture media containing 10% FBS (as a chemoattractant) was added. Cells were then treated with either ethanol or OST-01 and incubated for 72 hours. After incubation, non-invading cells and the ECM gel layer were removed using a cotton-tipped swab and washed with PBS. The invasive cells on the lower surface of the membrane were stained by dipping the inserts in the staining solution provided in the kit for 20 minutes, followed by rinsing with water and air-drying.

**Anoikis resistance assay**

Cells (1 × 10⁵) were prepared in 500 µL of culture media supplemented with anoikis-enhancing reagents (CytoSelect™ 24-Well Anoikis Assay, Cat# CBA-080). The cells were then added to each well of either the Anchorage Resistant Plate or a control plate. Following this, cells were treated with either ethanol or OST-01 for 24 hours. After incubation, photos were taken, and the cells were subjected to an MTT assay to quantify viable cells.

**Wound healing assay**

Cells were cultured in a 6-well plate until they reached 100% confluency. Wounds were generated by scratching the cell layer using a 1000 µL pipette tip. Following the wound creation, cells were treated with either ethanol or OST-01. Images of the cell culture were captured using a microscope at 0 and 48 hours. The scratch area was measured using ImageJ software to assess cell migratory capacity.

**Spheroid culture assay**

MDA-MB-231 cells were prepared at a density of 10,000 cells/200 µL and seeded in a U-bottom 96-well plate with a cell-repellent surface to form spheroids. The spheroids were treated with either ethanol or OST-01 for 24 hours. After treatment, they were stained with Calcein-AM and EthD-1 for 30 minutes at room temperature. Confocal images were then taken to visualize the stained spheroids.

**DNA fragmentation assay**

OST-01 or vehicle-treated cells were lysed on ice for 60 min in 500 μL lysis buffer containing 0.02% SDS, 1% Nonidet P-40 and 0.2 mg/mL proteinase K in PBS. Genomic DNA was extracted using the phenol/chloroform method. The pellet was dissolved in 50 μL of TE buffer (supplemented with 10 mg/mL RNase) for 2 h at 37 °C. A total of 10 μg of DNA was loaded on a 2% agarose gel and visualized under UV light.

**RNA-seq and Gene set enrichment analysis (GSEA)**

For high-resolution genomic profiling (mRNA-seq), sequencing was performed on an Illumina Hiseq 2500. RNA reads were trimmed to remove poly(A) tails and Illumina adapters using Trimmomatic, then aligned to the transcriptome from the GRCh38.p14 RNA sequences using Bowtie2 v2.5.1 under default settings. Gene expression levels were quantified using RSEM v1.3.3. Data normalization and inter-group comparisons to identify differentially expressed genes (DEGs) were conducted using Bioconductor packages, DESeq2 v1.40.2 and edgeR v4.2.2. Gene Set Enrichment Analysis (GSEA) v4.3.2, based on MsigDB v7.0 as the resource of annotated gene sets, identified groups of genes that share a common biological function or pathways. Also, gene ontology (GO) terms that are significantly enriched in a list of DEGs were identified.

**Immunoblotting analysis**

Cells were washed and harvested in ice-cold PBS and subsequently lysed in RIPA buffer containing 10 mM protease inhibitor cocktail (Thermo Scientific). For immunoblotting, 50 µg of each cell lysate was separated on NuPAGE 4-12% gradient gels (Invitrogen) and immunocomplexes were visualized with enhanced chemiluminescence reagent (Thermo Scientific). The list of antibodies is presented in Supplementary Table S1.

**Immunocytochemistry**

Cells were collected, washed in ice-cold PBS and mounted on glass slides using a Cytocentrifuge (CytoSpin4, 600 rpm, 10 minutes). Cells were then washed with PBS, fixed in 4% paraformaldehyde for 15 minutes and permeabilized in 0.5% Triton X-100 for 15 minutes. Non-specific epitopes were blocked with 5% bovine serum albumin (BSA) for 30 minutes. Primary antibodies are listed in Supplementary Table S1. Secondary anti-mouse/rabbit/goat-Alexa 594/488/647 goat antibodies were purchased from Thermo Scientific. Cell images were acquired using a Zeiss confocal laser-scanning-microscope (Zeiss LSM 800). Nuclei were counterstained with ProLong Gold Antiface with DAPI (Molecular Probes, Invitrogen).

**Annexin-V staining**

The Annexin-V and DAPI double staining method was used to evaluate apoptosis by flow cytometry. Cells were harvested and washed twice with Annexin-V binding buffer (BD Bioscience) and resuspended in 100 μL of the same buffer containing Annexin-V APC (BD Bioscience). Cells were then incubated in the dark at room temperature for 15 min, washed again and resuspended in 300 μL of buffer. DAPI (Sigma-Aldrich) was added immediately prior to analysis with a LSR II flow cytometer (BD Bioscience).

**Flow cytometry**

Cells were harvested into 15 mL tubes and washed with PBS. They were then pelleted and resuspended in FACS buffer (PBS containing 1% FBS and 0.25 mM EDTA). For apoptosis assays, cells were stained with Annexin V (eBioscience™ Annexin V Apoptosis Detection Kit, Cat#: BMS500FI-100, ThermoFisher) and 0.1 µg/mL DAPI. To evaluate stemness and differentiation, anti-human CD44 (APC/Fire™ 750, Cat#338818, BioLegend) and anti-human CD24 (PE/Cyanine7, Cat#311120, BioLegend) antibodies were used. For assessing ferroptosis, BODIPY™ 665/676 (Cat#B3932, Invitrogen) was employed.

**Densitometry quantification of Western Blot**

For quantification of protein expression from Western Blot results, the blots were measured using GeneSys program from Syngene, and the intensity was normalized to Actin expression. The expression level of the control was set to 1.0-fold, and the densitometry of treatments was compared to the control.

**Supplementary References**

1. Kang H, Hoang DH, Valerio M, Pathak K, Zhang L, Buettner R, et al. OST-01, a natural product from Baccharis coridifolia, targets c-Myc-dependent ribogenesis in acute myeloid leukemia. Leukemia. 2024;38(3):657-62.

2. Zhang J, Zhang X, Zhang L, Zhou F, van Dinther M, ten Dijke P. LRP8 mediates Wnt/β-catenin signaling and controls osteoblast differentiation. Journal of Bone and Mineral Research. 2012;27(10):2065-74.

3. Noh KT, Cha GS, Kang TH, Cho J, Jung ID, Kim KY, et al. Enhancement of paclitaxel-induced breast cancer cell death via the glycogen synthase kinase-3β-mediated B-cell lymphoma 2 regulation. BMB Rep. 2016;49(1):51-6.

4. Li W, Jin G, Zhou H, Gao Y, Ge Y, Zhang H. Exosome-transported circ_0001955 as a potent driver of breast cancer by regulating the miR-708-5p/PGK1 axis. Thorac Cancer. 2024;15(35):2486-99.

5. Hedayat M, Khezri MR, Jafari R, Malekinejad H, Majidi Zolbanin N. Concomitant effects of paclitaxel and celecoxib on genes involved in apoptosis of triple-negative metastatic breast cancer cells. Med Oncol. 2023;40(9):263.

6. Eiro N, Fraile M, Escudero-Cernuda S, Sendon-Lago J, Gonzalez LO, Fernandez-Sánchez ML, et al. Synergistic effect of human uterine cervical mesenchymal stem cell secretome and paclitaxel on triple negative breast cancer. Stem Cell Res Ther. 2024;15(1):121.

7. Deng S, Krutilina RI, Hartman KL, Chen H, Parke DN, Wang R, et al. Colchicine-Binding Site Agent CH-2-77 as a Potent Tubulin Inhibitor Suppressing Triple-Negative Breast Cancer. Mol Cancer Ther. 2022;21(7):1103-14.

8. Deng S, Krutilina RI, Wang Q, Lin Z, Parke DN, Playa HC, et al. An Orally Available Tubulin Inhibitor, VERU-111, Suppresses Triple-Negative Breast Cancer Tumor Growth and Metastasis and Bypasses Taxane Resistance. Mol Cancer Ther. 2020;19(2):348-63.

9. Mekonnen N, Yang H, Rajasekaran N, Song K, Choi YL, Shin YK. Indirect targeting of MYC and direct targeting in combination with chemotherapies are more effective than direct mono-targeting in triple negative breast cancer. Transl Oncol. 2025;51:102204.

10. Sriramulu S, Thoidingjam S, Siddiqui F, Brown SL, Movsas B, Walker E, et al. BUB1 Inhibition Sensitizes TNBC Cell Lines to Chemotherapy and Radiotherapy. Biomolecules. 2024;14(6).

11. Perona R, Esteve P, Jiménez B, Ballestero RP, Ramón y Cajal S, Lacal JC. Tumorigenic activity of rho genes from Aplysia californica. Oncogene. 1993;8(5):1285-92.

12. Mhaidat NM, Bouklihacene M, Thorne RF. 5-Fluorouracil-induced apoptosis in colorectal cancer cells is caspase-9-dependent and mediated by activation of protein kinase C-δ. Oncol Lett. 2014;8(2):699-704.

**Supplementary Table S1. List of antibodies used for IB and IF analysis**

| No | Antibody name | Information |
| --- | --- | --- |
| 1 | Anti-PARP antibody | Cat# 9542, Cell Signaling |
| 2 | Anti-PCNA antibody | Cat# sc-56, Santa Cruz |
| 3 | Anti-LRP8 antibody | Cat# MABN1872, Millipore Sigma |
| 4 | Anti-RAD54L antibody | Cat# ab11055, Abcam |
| 5 | Anti-ACTIN antibody | Cat# sc-47778, Santa Cruz |
| 6 | Anti-STC1 antibody | Cat# sc-293435, Santa Cruz |
| 7 | Anti-SDC1 antibody | Cat# sc-12765, Santa Cruz |
| 8 | Anti-SRSF3 antibody | Cat# 51039, Cell Signaling |
| 9 | Anti-GPX1 antibody | Cat# sc-133160, Santa Cruz |
| 10 | Anti-GPX4 antibody | Cat# sc-166120, Santa Cruz |
| 11 | SELENO R antibody | Cat# sc-135558, Santa Cruz |
| 12 | SELENO N antibody | Cat# sc-365824, Santa Cruz |
| 13 | SELENO M antibody | Cat# sc-514952, Santa Cruz |
| 14 | SELENO S antibody | Cat# sc-365498, Santa Cruz |
| 15 | SELENO P antibody | Cat# sc-376858, Santa Cruz |
| 16 | CD44 antibody | Cat# sc-9960, Santa Cruz |
| 17 | CD24 antibody | Cat# sc-19585, Santa Cruz |
| 18 | FN1 antibody | Cat# sc-271098, Santa Cruz |
| 19 | VIMENTIN antibody | Cat# sc-6260, Santa Cruz |
| 20 | CK19 antibody | Cat# sc-376126, Santa Cruz |

**Supplementary Figure Legends**

**Figure S1. Effects of OST-01 on proliferation and apoptosis in human cancer cell lines. A** Indicated cancer cell lines were treated with increasing doses of OST-01 for 24 hours. Left, heatmap showing cell proliferation levels measured by WST-1 assay. Right, heatmap displaying apoptosis levels measured by Annexin V staining. **B** Effects of OST-01 on proliferation in TNBC cell lines. A triple-positive BC cell line (BT474) and four TNBC cell lines [MDA-MB-231 (MB-231), MDA-MB-468 (MB-468), BT549, and 4T1] were treated with OST-01 in a dose-dependent manner for 24 hours. Cell proliferation was assessed using the WST-1 assay, and IC50 values for each cell line are shown. **C** Comparative effects of OST-01 and other therapeutic drugs on 4T1 and MDA-MB-231 TNBC cell lines and the triple-positive BT474 cell line. Cells were treated with 1 µL/mL of OST-01, 2 µM of Taxol, or 5-FU for 24 hours. Apoptosis was measured by Annexin V staining.

**Figure S2. Effects of OST-01 on oncogenic activities in TNBC cells in vitro.** MDA-MB-231 TNBC cells were treated with 0.5 µL/mL of ethanol control or OST-01 for 24 hours. **A** Colony Formation Assay: Representative images of colonies formed by cells treated with ethanol control or OST-01. **B** Anoikis Resistance Assay: Representative images of cells under anoikis conditions treated with ethanol control or OST-01. **C** Wound Healing Assay: Representative images showing wound healing in cells treated with ethanol control or OST-01. **D** Invasion Assay: Representative images of invasion levels in cells treated with ethanol control or OST-01. **E** MDA-MB-231 cells (10^4^ cells per well) were cultured in 3D to generate spheroids. Spheroids were then treated with 0.5 µL/mL of ethanol control or OST-01 for 24 hours and stained with Calcein-AM (green, indicating live cells) and EthD-1 (red, indicating dead cells). Left, representative confocal image of MDA-MB-231 spheroids stained with Calcein-AM and EthD-1 is shown. Right, quantification of Calcein-AM and EthD-1 fluorescence signals (n=10).

**Figure S3. Effects of OST-01 on LRP8 signaling in TNBC cells. A-B** TNBC cells (4T1 and MDA-MB-231) or BT474 triple-positive BC cells (3 × 10⁶ cells each, n=3) were treated with 1 µL/mL of ethanol control or OST-01 for 24 hours. mRNA was extracted for RNA sequencing. **A** Unsupervised hierarchical clustering reveals significant changes in gene expression, represented in the heatmap. **B** The results of gene set enrichment analysis are shown in dot plot with FDR corrected statistical significance. **C** Top 20 downregulated genes in TNBC cells (4T1 and MDA-MB-231) or BT474 triple-positive BC cells treated with OST-01 compared to ethanol-treated control. **D** Volcano plot showing 7 upregulated and 7 downregulated genes in MDA-MB-231 TNBC cells treated with OST-01 compared to ethanol-treated controls. **E** Effects of OST-01 on the expression of key regulators of BCSC. 4T1 TNBC cells (left) and BT474 triple-positive BC cells (right) were treated with 1 µL/mL of ethanol control or OST-01 for 24 hours. mRNA levels were measured by qPCR.

**Figure S4.** **Effects of OST-01 on LRP8-regulated ferroptosis in TNBC cells. A** Coessentiality network analysis for OST-01-regulated LRP8 in MDA-MB-231 TNBC cells, performed using FIREWORKS (https://mendillolab.shinyapps.io/fireworks/). Five primary modules are identified: (1) selenoproteins, (2) selenocysteine metabolism, (3) glutathione, CoA, and NAD metabolism, (4) ferroptosis, and (5) LRP8-regulated proteins. The thickness of the lines represents Pearson correlation. **B** Gene ontology (GO) analysis with all genes in the network analysis in **A**.

**Figure S5.** **Effects of OST-01 on the expression of LRP8 and selenoproteins in TNBC cells. A** MDA-MB-231 TNBC cells were treated with 1 µL/mL of ethanol control or OST-01 for 24 hours. The treated cells were stained with the indicated antibodies, and fluorescence images were captured using a confocal microscope. Scale bar, 10 µm. **B** Effects of OST-01 on SDC-1, LRP8, and GPX4 expression in TNBC tumors. MDA-MB-231 xenograft tumors isolated from mice treated with ethanol control or OST-01 (as described in **Fig. 1E**) were subjected to frozen sectioning and stained with the indicated antibodies. The images were captured using confocal microscopy. Scale bar, 1 µm.

**Figure S6. Effects of OST-01 on mesenchymal-epithelial transition in TNBC cells.** Gene set enrichment analysis (GSEA) graphs show the impact of OST-01 treatment on mesenchymal-epithelial transition markers. The graphs illustrate enrichment scores for genes associated with the downregulation of Epithelial-Mesenchymal Transition (left), Breast Cancer Luminal vs. Mesenchymal transition (middle), and Breast Cancer ESR1 upregulation (right) in 4T1 TNBC cells (**A**), BT474 triple-positive breast cancer cells (**B**), and MDA-MB-231 TNBC cells (**C**). Cells were treated with 1 µL/mL of ethanol control or OST-01 for 24 hours. ES: Enrichment Score, NES: Normalized Enrichment Score, FDR: False Discovery Rate.

**Figure S7.** **Raw Western Blot data.** **A** Raw Western Blot data corresponding to **Fig.1B**. **B** and **C** Raw Western Blot data corresponding to **Fig. 2A**. **D** and **E** Raw Western Blot data corresponding to **Fig. 2D**. **F** Raw Western Blot data corresponding to **Fig. 2E**. **G** Raw Western Blot data corresponding to **Fig. 2I**.
